# Supplementary material for: Are carbon emissions trading and green financial instruments synergistic? -Comprehensive quantitative research based on content analysis
Source: PLoS One. 2024 Mar 7;19(3):e0298601. doi: 10.1371/journal.pone.0298601 (PMC10919601; doi:10.1371/journal.pone.0298601)
Supplement: S1 Data — (ZIP) [file pone.0298601.s001.zip › Data/地级市Z_F工作报告.zip!/─■╧─/╬Γ╓╥╩╨/└┤╫╘╣1⁄2╓┌║┼í╛┬φ┐╦╩2╛▌═°í┐_2008.docx]

各位代表：

现在，我代表市人民政府向大会作政府工作报告，请予审议，并请市政协委员和其他列席人员提出意见。

一、2004年以来的工作回顾

面对市情的新变化、发展的新要求，在自治区党委、政府和市委的领导下，在市人大和市政协的监督支持下，政府工作坚持以科学发展观统领全局，紧紧依靠全市各族人民，凝心聚力谋发展，开拓创新求突破，圆满完成了市二届人大一次会议确定的各项目标任务。全市地区生产总值由2003年的82.6亿元增加到136亿元，年均增长12.7%，人均生产总值迈上1000美元新台阶。地方财政收入由2003年的4.7亿元增加到13亿元，年均可比增长14.9%。城镇居民人均可支配收入和农民人均纯收入分别由2003年的6126元和2507元增加到9680元和3400元，年均分别增长12.1%和7.9%。市域经济综合实力稳步提升，县域经济活力明显增强，青铜峡市跃居西部百强县第43位，盐池县在全区山区县中率先跨过财政收入亿元关口。

新型工业基础夯实，工业主导地位更加稳固。全市工业总产值由2003年的109.8亿元增加到233亿元，翻了一番，年均增速达15.6%。能源化工、机械制造、风力发电、建筑建材、农产品加工等产业不断壮大，骨干企业市场竞争力大幅提高，全市规模以上工业总产值、增加值分别达到208.4亿元和56.3亿元，比2003年增长1.3倍和1.18倍。全市销售收入过亿元的企业由8家发展到21家，有4家企业产值超过10亿元，12家企业进入自治区重点工业骨干企业行列。全市6个工业园区入园企业达到249家，完成产值180亿元。启动实施了太阳山移民开发区建设，被自治区确定为推动宁夏经济社会跨越式发展的又一重要增长极，累计完成投资24亿元，太阳山供水工程和庆华集团一期100万吨煤焦化等一批重点项目顺利建成，一座新型现代工业城正在崛起。循环经济加快发展，节能减排初见成效。地方工业万元增加值能耗低于3.37吨标准煤、水耗低于1380吨，固体废物利用率达到50.49%。依法关闭了23家小造纸企业。金昱元集团进入全国首批46家循环经济试点企业行列。

“三农”工作不断加强，新农村建设健康发展。全市农业总产值由2003年的26.8亿元增加到41.9亿元，年均增长5.6%。粮食生产稳定增长，奶产业、设施农业、酿酒葡萄、高酸苹果、滩羊、肉牛、圆枣、甘草等特色产业显示出强大的发展后劲。全市奶牛存栏达到18万头，比2003年增长了1.8倍。利通区被评为“全国牛奶生产强县”。培育市级以上农业产业化龙头企业77家，发展农村专业经济合作组织253个，认定无公害产地60个。“盐池滩羊”产地证明商标成功通过国家注册。实施村企互动共建新农村活动及千村整治、塞上农民新居、山区危房危窑改造等工程，全市新建塞上农民新居示范点20个，完成危房危窑改造5853户，农村生产生活条件有效改善。市区率先在全区开展试点，农村面源污染综合整治取得显著效果。土地、矿产资源、水资源节约利用取得新成效，耕地保护、水利建设、水土保持不断加强。全市砌护渠道600多公里，新建改造水利设施8000多座，新修“三田”18.8万亩，改造中低产田35.9万亩，解决了22万人的安全饮水问题。造林绿化、封山禁牧、退耕还林(草)取得丰硕成果，累计完成人工造林面积196.2万亩，封山育林33.5万亩，草原围栏488万亩，森林覆盖率由2003年的6.7%提高到9%。青铜峡市跻身全国林产业示范县和绿色小康县行列，盐池县荣获全国生态建设先进县。扶贫开发成效显著，抗旱避灾农业与封山禁牧接续产业稳步发展，整村推进工程、同心东部引水工程等深入实施，市区孙家滩、同心惠安新村等生态移民项目发挥效益，引领作用明显。贫困人口由2003年的17.1万人减少到了2.3万人。

基础建设持续加快，城乡面貌显著变化。全社会固定资产投资累计完成241.5亿元，年均增长22.9%。开工建设了一批重点基础设施项目。全市新建等级公路1023公里，公路通车里程比2003年增加1349公里。太中银铁路过境段、盐中高速公路过境段等项目顺利推进。市区实施了老城商贸核心区改造、新区开发、城中村改造、城乡环境综合整治与绿化美化等工程，城市功能和品位不断完善和提升。城市建成区面积由16.7平方公里扩展到23平方公里，人均公共绿地面积由0.6平方米增加到7.5平方米。加快了城市西移与近水亲河，滨河大道、金滨路建设，防洪排涝工程启动实施，大幅拉开了滨河生态城市框架。县城建设各具特色、各展异彩，道路、供热、供水、垃圾处理等基础设施日趋完善。青铜峡小坝东区开发、城市景观绿化，盐池瓮城古迹修复、革命历史纪念馆工程，同心北沟综合治理、红军西征纪念园建设，红寺堡城区北扩、生态绿化等一批项目顺利实施，城市面貌焕然一新，人居环境不断改善，集聚效应和辐射带动能力日益增强。

第三产业加快发展，商贸流通彰显活力。第三产业增加值预计达40.8亿元，年均增长9.7%。 “沙漠王子”等品牌大步走向市场，成为全国知名品牌。城乡流通体系不断完善，物价基本保持稳定，消费市场更加繁荣兴旺。连锁经营、物流配送、电子商务、信息中介、金融、保险、证券、社区服务、餐饮等服务业快速发展。全市社会消费品零售总额由2003年的18.9亿元增加到30亿元，年均增长12.9%。“万村千乡市场工程”加快推进，全市建成标准化农家店650个，受益农民达65万人。红色经典、生态农业等特色旅游资源开发加快，旅游市场日趋活跃，旅游总收入由2003年的4800万元增长到3.5亿元，增长6倍多。盐池、同心进入全国百家红色旅游经典景区行列。

改革深化有序推进，对外开放不断扩大。企业产权制度改革取得新的进展，企业改制面达95%。完成了国有企业下岗失业人员基本生活保障向失业保险制度并轨工作。行政管理体制改革稳步推进，平稳实施了调整市区机构设置、进一步理顺市区管理职能的决定和全市党政群机关编制实名制管理。完成了市区经营性事业单位转企工作。财政收支分类改革不断深化，部门预算管理得到加强，国库集中支付和乡财县管乡用制度全面建立，政府采购规模逐步扩大，收入分配政策和规范津补贴工作得到及时落实。撤并乡镇与农村税费改革顺利完成，取消了农业税，累计化解乡镇债务15175万元。农村义务教育经费保障机制改革全面推进。辖区金融体制改革不断深化，组织体系日益健全，税收征管体制更加完善，金融、税收对经济社会发展贡献明显提升。成功举办了“中国文化宣传周”、“全国中小学生书法节”等大型活动，大幅度提升了吴忠的知名度。主动“走出去”、“请进来”开展招商引资和经贸合作。先后组团到发达省市山东、浙江、江苏、北京等地考察学习，专门组团赴马来西亚，积极探寻与马来西亚及中东国家发展经贸关系的新途径，迈出了合作共赢的实质性步伐。引进了中国华能、内蒙庆华、中国阳光等一批大企业、大集团落户吴忠，与湖南长沙、中国农业大学、宁夏大学等地区与机构建立了友好合作关系，实施了一批合作项目。四年累计落实招商引资合同项目220个，实际到位资金91亿元。出口企业发展到83家，今年预计完成进出口总额1100万美元，是2003年的1.3倍。

科教文卫蓬勃发展，社会事业协调推进。科技事业发展步伐加快，科技特派员创业行动成效显著，以企业为主体的创新体系逐步建立，一大批新技术、新成果得到推广应用，宁夏吴忠国家农业科技园区通过自治区验收，全国科技强警示范城市创建任务基本完成，科技进步对经济增长的贡献率达到47%。教育优先发展的地位更加突出，全社会办教育的氛围更加浓厚，全市提前一年完成“两基”攻坚任务，利通区、青铜峡市实现教育强区(市)目标。信息技术教育居自治区领先水平。教育资源有效整合，学校布局不断优化，实施中小学新改扩建项目200个，创建自治区“百标学校”24所。吴忠职业技术学院更名为宁夏民族职业技术学院，青铜峡市职业教育中心被列为全国重点职业中专。群众性精神文明创建活动广泛开展，建设村级组织活动场所116个，创建全区五星级社区3个，社会文明程度不断提高，涌现了全社会崇尚的金峰、魏斌、朱玉等英雄模范人物，学先进、比奉献的氛围更加浓厚。文化体育事业日益繁荣，群众文化丰富活跃，宣传文化体育中心户创建经验在全区推广，吴忠体育馆等一批文体设施建成使用，成功举办了太阳山之夜大型交响音乐会、吴忠市首届运动会、中西部十二省市区领导干部乒乓球比赛等大型文体活动。文物保护和开发得到加强，董府、盐池张家场古城址和青铜峡鸽子山遗址被列为全国重点文物保护单位。城乡广播电视公共服务体系基本建立，完成有线电视数字化平移，微波信号覆盖全市农村地区。公共卫生和医疗服务体系建设不断加强，市疾控中心等14个市(县)级医疗卫生机构、11个城市社区卫生服务站、40所乡镇卫生院和547个标准化村卫生室投入使用。新型农村合作医疗实现全覆盖，农民参合率达到83%以上。药品统一招标、统一价格、统一配送“三统一”制度全面实行。吴忠市被评为“全国无偿献血先进城市”。农民健康教育与健康促进行动工程启动实施。青铜峡市创建为国家食品安全示范县。人口与计划生育工作成效显著，人口自然增长率首次攻克千分之十难关，今年控制在9.73‰以内，计划生育率达到92.53%。民族宗教政策深入落实，宗教稳定，民族团结。外事侨务、气象、档案、住房公积金等工作取得新成绩，妇女儿童、残疾人、老龄、统计、地震、保密等工作全面推进。国防教育和人防、民兵预备役等工作不断加强，国民经济动员正规化建设走在全区前列，双拥共建取得新成效，吴忠首次获得国家双拥模范城称号。

人民生活水平稳步提高，民生问题得到大力改善。就业再就业工作力度加大，四年实现新增城镇就业4.5万人，帮助6388名失业人员实现再就业，城镇登记失业率一直控制在4.2%左右。社会保障体系逐步完善，城镇养老、医疗、失业、工伤、生育保险参保人数达到24万人。为38家国有关闭破产和困难企业3907名职工办理了医疗保险，市区5家农牧场3600多名职工全员进入养老保险，解决了多年想做而未做成的疑难问题。城乡低保制度不断完善，社会救助体系逐步健全，3.68万名城镇居民享受最低生活保障，5.28万名农村特困人口得到低保救助，101户城镇低收入家庭住上廉租房，1239户农村残疾人住房困难得到解决。新改扩建敬老院9所，五保集中供养率达到40%以上。城乡居民住房条件明显改善，城镇和农村人均住宅面积分别达到27.3平方米、27.4平方米，比2003年分别增加7平方米和2.1平方米。着力保护人民群众正当权益，近几年仅市区就为农民工清欠工资2145.9万元，财政筹集兑付拆迁安置补偿资金15852万元，清理偿还历年欠款17681万元。建立健全了救灾工作体系，四年累计投入救灾资金8700万元。优抚政策全面落实，抚恤补助标准自然增长机制初步建立。

政府自身建设不断改进，公共服务切实增强。高度重视政府管理创新和效能建设，不断深化行政审批制度改革，四年清理各类授权行政许可事项263项，取消63项。整合提升市、县政府政务大厅职能，实行行政审批事项集中办理。全面推行政务公开，强化监察和审计监督，开展商业贿赂专项整治。制定实施政府工作规则，成立政府法律顾问团，完善公众参与、专家论证和集体决策相结合的机制，政府决策更加科学民主。严格执行市人大及其常委会的决议、决定，及时向政协通报政情，认真办理人大代表议案、建议和政协委员提案，与民主党派、工商联的沟通联系继续加强，与工会、共青团、妇联等群众团体的联系更加密切。民主法制建设不断深入，完成“四五”普法，启动“五五”普法。加强制度建设，市政府及其组成部门新订或修改出台了各类制度300多项，规范了运行程序，增强了服务效能。大力推进“平安吴忠”建设，建立重大突发公共事件应急机制。市场经济秩序有了较大改观，产品质量、食品药品、安全生产等监管不断加强，安全生产控制指标降幅连续三年居全区前列。认真做好信访工作，积极调处人民内部矛盾。加强社会治安综合治理，严密防范和依法打击各类违法犯罪活动，保持了社会稳定。

这些成绩的取得，是在自治区党委、政府和市委正确领导下，全市各族人民锐意进取、干事创业的结果，也是全市上下和衷共济、通力协作、共同奋斗的结果。在此，我代表市人民政府，向全市各族人民，向各民主党派、工商联和各人民团体，向离退休老同志，向中央和自治区驻吴各单位，向公安干警和驻吴人民解放军、武警官兵，向所有关心、支持吴忠发展的社会各界朋友，表示崇高的敬意和衷心的感谢！

各位代表，本届政府履行职责四年以来，深刻认识和正确把握新市情带来的新机遇，面对矛盾与困难、压力与挑战，坚持谋长远、打基础，探索新思路、寻找新突破；始终把关系民生的事放在各项工作的首要位置，财力再紧再难，都坚持公开承诺为民办实事，坚持优先解决教育、医疗、安全饮水、弱势群体救助、失地农民安置补偿等问题，从点点滴滴着手为百姓谋福祉，从方方面面努力让群众得实惠；紧扣山川发展差别大的实际，在推动川区快速发展的同时，更加关注山区的发展，更加重视山区群众的脱贫致富；高度维护市委的权威，自觉维护与人大、政协及社会各界的团结；始终维护民族团结，坚决保持宗教稳定，充分调动一切积极因素，形成加快发展的强大合力；以更加开放的理念，举办好提升吴忠知名度的各项重大活动，采取一系列打响吴忠品牌的有效措施，让吴忠大步走向国际。这些都为全面推动我市经济社会健康快速发展注入了新的活力和动力。也可以说，这四年是我们攻坚克难、负重拼搏、再求突破的四年；是吴忠积蓄实力、重新崛起、阔步前进的四年；是全市人民奋起直追、艰苦创业、享受更多实惠的四年；也是吴忠城乡面貌显著变化、各行各业展现新姿的四年！

成绩来之不易，但回顾总结过去，豪迈中我们感受更多的是压力和责任，是对困难与不足的审视和警觉。应当看到，吴忠的发展仍然面临着不少困难和问题。主要是：市域经济总量小、实力弱，山川之间、城乡之间发展不平衡；经济增长方式较为粗放，资源、能源消耗较高，节能减排、环境保护压力大；工业结构性矛盾突出，企业自主创新能力不强；现代农业发展不快，山区扶贫开发任务繁重；农民增收渠道单一，收入增长幅度不高；财政保障能力较弱，硬性支出加大；困难群众生活救助、失地农民转移就业等一些关系民生的问题尚未得到有效解决，医疗、教育等公共产品供给还难以充分满足人民群众的需求；政府效能建设有待加强，发展环境需要不断改善；招商引资的成效还不突出，引进的大项目还不多。对这些问题，我们必须以高度负责的精神，切实加以解决，决不辜负人民群众的期望。

二、今后五年的目标任务和总体要求

目前，吴忠仍然处在一个攻坚克难、爬坡追赶的发展阶段，经济欠发达是最大的市情，推进跨越式发展是吴忠的必然选择。从现在到2012年，是全市上下必须积极应对挑战、抢抓机遇，推进跨越式发展的关键五年。我们必须从战略上，牢牢把握解放思想这一法宝，不断破除束缚发展的思想、体制、制度障碍，努力营造开放包容、借力发展、争上项目的创业氛围，让创业活力更加喷涌迸发；牢牢把握科学发展这一灵魂，切实转变发展方式，加快经济结构调整和产业培育，努力实现速度质量效益相协调，消费投资出口相协调，人口资源环境相协调；牢牢把握构建和谐这一根本，统筹城乡、山川发展，更加注重改善民生，切实维护公平正义，激发社会活力，让改革发展成果惠及广大人民群众；牢牢把握改革创新这一主线，更加注重观念创新、制度创新、科技创新和工作创新，提高全社会自主创新能力，全力推进我市的改革开放和现代化事业。

今后五年政府工作的总体要求是：高举中国特色社会主义伟大旗帜，认真贯彻党的十七大、自治区第十次党代会和吴忠市第三次党代会及三届四次全体(扩大)会议精神，解放思想、开拓创新，深化改革、扩大开放，着力转变发展方式，大力实施兴工强市战略和城镇化带动战略，加快推进现代农业和服务业发展，全面加强社会建设、文化建设和民主政治建设，为建设山川秀美、特色鲜明、民族团结、社会稳定、人民富裕的小康吴忠、实力吴忠、魅力吴忠、和谐吴忠而努力奋斗。跨越式发展的奋斗目标是：力争到2012年，全市地区生产总值达到270亿元，在今年基础上翻一番；地方财政收入20亿元以上；全社会固定资产投资累计超过700亿元；城镇居民人均可支配收入、农民人均纯收入分别达到15000元和5000元；人口自然增长率控制在10‰以内；万元GDP能耗下降20%，主要污染物排放总量削减30%。

未来五年，必须在兴工强市上迈出更大步伐。坚定不移地实施兴工强市战略，围绕“两城五园”建设，加快资源开发、结构调整、项目带动和技术创新。大力培育以煤炭、电力为主的能源产业集群，以煤化工、石油化工为主的化工产业集群，以铝、镁及其深加工为主的新材料产业集群，以造纸包装、农产品加工、羊绒深加工等为主的轻工产业集群；做强以仪器仪表、重型装载机械为主的制造业、以多晶硅冶炼和深加工为主的高新技术产业产业。全力打造太阳山新型能源化工城，重点发展煤炭、电力、化工、新材料及新能源产业，建成占到全区三分之一份额的煤化工基地，成为全区镁合金等新材料生产研发基地、大型风电场和太阳能研发利用基地，到2012年GDP超过50亿元；打造青铜峡新型铝业城，大力发展铝、镁、镁合金及其精深加工产业，建成西部重要的铝、镁及其合金等新材料加工基地，到2012年GDP超过90亿元。加强市区金积、盐池东顺、同心羊绒、青铜峡嘉宝和红寺堡等五个工业园区的基础设施建设，完善配套功能，优化发展环境，积极承接发达地区产业转移，引导现有企业向园区聚集，使园区真正成为产业聚集、特色鲜明、带动县域经济发展的重要增长极。力争把金积工业园建成中西部重要的乳品加工基地、造纸印刷工业基地；把青铜峡嘉宝工业园建成市域重要的机械加工和农产品加工基地；把盐池东顺工业园建成市域重要的石化产业基地；把同心羊绒工业园建成全国重要的羊绒集散和加工基地；把红寺堡工业园建成全区以葡萄深加工为主的农产品加工基地。通过产业培育和园区建设，巩固提高中小企业发展，引进建设一批新的项目，使地方工业的实力更加强大，对地方财政的贡献更加突出。到2012年，全市工业总产值突破500亿元，在现有基础上翻一番，对经济增长、财政收入的贡献率分别达到70%和65%。年销售收入过亿元的企业发展到50家、过10亿元的企业发展到13家、过50亿元的企业发展到4家。

未来五年，必须在推进城乡一体化上迈出更大步伐。深入实施城镇化带动战略，统筹布局全市城镇基础设施建设，大步推进城乡一体化进程。加快建设覆盖全市、贯通内外的综合道路交通网络。规划建设河东机场至太阳山、古窑子至青铜峡、滚泉至桃山口、沿山高速四条穿境高速公路及吴忠黄河公路大桥、小坝至青铝集团沿河快速通道、S101线市区至金积拓宽改造等重大道路交通项目，在吴忠市区形成以主城区为轴心、周边5公里为半径的绕城高速圈。进一步加快城镇供排水、消防、供电、供热、供气、通讯、公交、环卫等公共设施建设，不断完善城市功能。依托区位条件、历史文化、民俗风情和产业定位，培育城市特色。力争5年内在城市交通、公用设施、生态景观建设和城市品牌塑造等方面迈出扎实步伐。市区着力打造商贸发达、特色浓郁的滨河生态城市。到2010年，实现市区与青铜峡小坝跨河相连、与金积及青铜峡工业区结成一体，城市建成区面积达到50平方公里，人口达到35万，绿化覆盖率达到40%。到2012年初步建成国家园林城市、全国卫生城市、环保模范城市，使吴忠成为更加适宜居住和创业、承接大银川、辐射陕甘蒙毗邻地区的宁夏次中心城市。青铜峡进入全国百强县；盐池建成宁夏山区工业第一县，争创自治区园林城市；同心成为干旱带扶贫开发示范县，建成具有浓郁塞上风情的生态商贸城市；红寺堡高标准完成移民扶贫开发任务，建成生态城市；太阳山成为宁夏经济社会发展的重要增长极，建成水保生态城市。建成一批布局合理、设施配套、功能健全、环境优美、富有特色的新型小城镇。

未来五年，必须在新农村建设上迈出更大步伐。紧紧围绕增加农民收入，加大农业投入，不断调整优化农业结构，落实支农惠农政策，全面发展农村经济，加快推进社会主义新农村建设。现代农业快速发展，特色产业规模持续扩大，建成100万亩优质粮食、50万亩高效节水设施农业、50万头奶牛、50万头肉牛、600万只滩羊、50万亩高酸苹果、50万亩葡萄、50万亩红枣、100万亩甘草、200万亩饲草等“十大农业产业基地”。保障粮食等农产品供给，巩固提高农民市场化组织程度与农业抗风险能力。培育壮大龙头企业与各类专业协会、营销组织，拉长产业链，提高农产品附加值。到2012年，川区率先建成全区现代农业示范区，山区率先建成全区节水旱作农业示范区。加快基础设施向农村延伸、公共服务向农村覆盖的进程。山区农村全部实现通路、通电、通邮、通电话、通班车、通广播电视，川区在此基础上，绝大多数通上自来水，使用沼气、太阳能等清洁能源，各乡建有垃圾集中填埋场、各行政村设立垃圾集中清收点、各自然村都有环境卫生保洁员。以“塞上农民新居”、“危窑危房改造”和村容村貌整治为抓手，大力实施中心村建设和农村小康环保行动计划，力争完成所有建制村的综合整治，使农村基础设施大幅度完善配套，农民人居环境和生活质量得到更大改善，基本形成富有现代气息的村庄体系。山区按规划完成生态移民工程，从根本上解决山区农民脱贫问题。通过新农村建设的加速推进，让更多的农民走上硬化路、喝上干净水、用上卫生厕、使上洁净能、住上砖瓦房。

未来五年，必须在发展第三产业上迈出更大步伐。采取更加积极的扶持政策，着力加快服务业发展。把旅游业作为新的经济增长点，挖掘整合大漠黄河、草原生态、红色经典等特色旅游资源，加大旅游项目策划、包装、宣传力度，完善提升景区服务配套功能，打造精品旅游线路和特色旅游景区，培育发展旅游商品等配套产业，增强吸引力，努力建成继沙湖、沙坡头之后宁夏又一特色精品旅游区，使吴忠成为宁夏乃至西北旅游目的地，旅游业对第三产业发展拉动作用显著增强。力争到2012年，全市接待游客达到220万人次，旅游总收入突破11亿元。充分发挥传统商贸优势，突出发展现代商贸流通业，以牛羊肉及其皮毛、废旧金属等可再生资源、粮油食品、瓜果蔬菜等为重点，集中建设一批基础配套、信息便捷、辐射面广的专业化集散市场，全面发展休闲、会展、中介、金融、信息等新兴业态，构建与周边城市功能互补的物流体系，把吴忠打造成比较优势明显的区域性商贸物流中心。

未来五年，必须在建设生态文明上迈出更大步伐。严格保护耕地，着力推进资源节约和高效、有序、有偿利用，在强化土地、矿产和水资源管理、加大节能减排、发展循环经济上取得新成效，重点行业、企业的能源、原材料、水资源和土地集约节约水平明显提高，重点流域、重点区域、重点行业环境质量明显改善，清洁生产模式广泛推行。基本实现工业污染物达标排放和循环利用，努力为子孙后代留一顶蓝天、一方清水和一片净土。继续实施退耕还林、退牧还草、封山禁牧和植树造林，加强小流域治理、防洪、防涝和防沙治沙，减少水土流失。充分开发利用市域内的湖泊、湿地、水系、公园等资源，加强河流沿线、主干道路两侧生态绿化和城镇景观绿化，构建城乡联动、山川共进的生态环境建设大格局。到2012年，新增造林面积320万亩，其中治沙130万亩，森林覆盖率达到15%，使全市草原、植被稳定恢复，山区林草相间，生态环境明显改善，再现山川秀美新景象；川区林网密布、沟渠纵横、湖泊湿地相连，凸现塞上江南好风光。

未来五年，必须在改革开放上迈出更大步伐。坚持社会主义市场经济的改革方向，正确处理改革、发展、稳定的关系，增强改革措施的协调性和针对性，形成有利于各类经济主体平等参与、公平竞争、有序发展的体制机制。继续深化企业产权制度改革，实现制度、管理、技术创新，使改制企业改彻底、有活力、快发展。积极创造条件，鼓励中小企业大发展，鼓励非公有制企业创新经营机制，推进民间资本与市外资本嫁接联合，大力发展家庭经营型企业，让非公有制经济成为市域经济主体，全民创业成为发展的主旋律，让更多群众拥有财产性收入。稳步推进行政体制改革，形成权责统一、分工合理、决策科学、执行顺畅、监督有力的行政管理体制。进一步加快科技、教育、文化、卫生、城市公用事业等领域的改革进程。注重信用建设，深化辖区银行业改革，不断健全金融服务体系。继续推进公共财政体制改革，不断加强财政、税收工作，培植壮大新的财源，提高地方财政实力，使财政公共服务职能明显增强，努力实现基本公共服务均等化。不断扩大对外开放，充分发挥土地、煤炭等资源优势，在引进战略投资、对接科研院所、扩大对外合作交流等方面取得实质性进展。进出口总量年均增长10%，招商引资实际到位资金年均增长20%以上。经济社会发展环境明显改善，对外知名度、地方吸引力、环境竞争力显著提升。

未来五年，必须在社会事业全面发展上迈出更大步伐。继续坚持教育优先发展战略，不断加大教育投入，提高教育发展水平，实现教育公平，实现城乡、山川教育均衡发展，实现创建教育强市目标。让农村学生能够享受与城市学生同等的教育资源，让城乡贫困家庭子女都能接受高中阶段教育。规划建设教育园区，争取将宁夏民族职业技术学院建成全区示范性高职院校，让初、高中毕业不能升学的学生都能接受职业技术教育和培训。加大科技投入，推进科技创新，以企业为主体、市场为导向、产学研相结合的技术创新体系基本建立，农业科技支撑能力明显提高，科技进步对经济增长的贡献率提高到53%。坚持把社会主义核心价值体系融入国民教育全过程，着力加强社会公德、职业道德、家庭美德、个人品德建设，抓好青少年思想道德教育，全民素质不断提高。加快发展和谐文化，培育文化产业，促进公益性文化事业更加繁荣，基本实现各乡镇有文化站、各行政村有文化室、各自然村有文化大院、城市社区有文体活动中心。推动医疗卫生事业快速发展，巩固新型农村合作医疗制度，适当提高政府补助标准；建立城市居民基本医疗保险制度，初步实现全民医保；加快推进城市社区卫生服务体系建设，完善农村三级卫生服务网络，市区办好一所高水平的综合医院和几所各具特色的专科医院，每个县市区集中办好一所能够满足群众正常就医需求的综合医院；深入推进农民健康教育与健康促进行动工程，实现城乡居民人人享有基本医疗卫生服务。全面推行农村计划生育奖励扶助制度和“少生快富工程”，依法控制山区人口增长，稳定川区低生育水平。以创业带动就业，统筹做好城镇新增劳动力就业、农村劳动力转移就业、下岗失业人员再就业工作，五年新增就业5.5万人，城镇登记失业率控制在4.2%以内。农村劳动力转移就业每年稳定在15万人以上，让越来越多的农村劳动力通过技能培训、转移就业转化为市民、商人、产业工人和新型农民。山区老百姓安全吃水问题基本解决。加快健全覆盖城乡的社会保障体系，城镇养老、失业、医疗、工伤、生育保险制度更加完善，农村养老保险和失地农民社会保险问题得到逐步解决，城乡困难群众最低生活保障水平稳步提高。加大城市廉租住房和经济适用房建设力度，确保城市低收入家庭住房困难得到应保尽保。全面贯彻党的民族宗教政策，依法加强对宗教事务的管理。民主法制、“平安创建”进一步加强，社会更加稳定和谐，人民群众更加安逸、更加幸福。

各位代表，实现未来五年的跨越式发展，是时代的召唤、人民的愿望。我们坚信，只要始终坚持在解放思想中统一思想、在改革开放中开拓进取，一年一个新变化，三年上个新台阶，五年有个大发展，就一定能够实现我市跨越式发展的宏伟目标，就一定能够开创建设小康吴忠、实力吴忠、魅力吴忠、和谐吴忠的崭新局面。

三、2008年工作建议

明年是全面贯彻十七大精神、加快推进吴忠跨越式发展的关键之年、起步之年。我们要以良好的精神状态和优异的发展成效，向自治区成立50周年和地级吴忠市成立10周年献礼。主要预期目标和约束性指标是：地区生产总值增长13%，地方财政收入可比增长16%，全社会固定资产投资增长25%、力争30%，社会消费品零售总额增长12%，城镇居民人均可支配收入增长10%，农民人均纯收入增长8%，人口自然增长率控制在10‰以内，城镇登记失业率控制在4.2%以内，二氧化硫削减6%，化学需氧量削减7%。

全面完成明年各项任务，要重点做好以下七个方面的工作：

(一)抓好“两城五园”建设，强力推进工业经济提速增效

以园区为平台，以项目为支撑，加快产业结构调整，做大工业经济总量。全市工业总产值、规模以上工业增加值均增长18%以上，地方工业增加值增长28%以上。要提升园区承载能力。按照“抓园区、盯项目、扩规模、提质量”的思路，加快建设“两城五园”，抓紧修编完善工业园区发展规划，加快园区体制和机制创新，加大基础配套和招商引资力度，完成园区基础设施投资7.8亿元，引进亿元以上的项目10个。要壮大优势特色产业。力争开工建设华能集团韦州综合利用电厂等项目，壮大能源产业；推进青铝集团15万吨铝板带等项目建设，做强新材料产业；力促庆华集团30万吨甲醇等项目建设，推动化工产业发展；实施银星能源多晶硅等项目，积极培育高新技术产业；加快万兴集团3000台载重汽车等项目建设，扩大制造业规模；完成新源纸业等5家造纸企业搬迁改造，抓好娃哈哈6万吨奶粉一期和夏进1.5万吨奶粉新生产线、祥福公司400吨羊绒纱等项目建设，推动轻工和农产品加工业发展提速。全市新增工业产值33亿元。要支持骨干企业做大做强。对全市利税前30名工业企业实行重点协调服务，支持骨干企业改造装备、开发产品、扩大销售。完成吴忠卷烟厂联合工房及配套设施技改项目，使卷烟生产能力达到15万箱。全市规模以上工业产值达到245亿元，增长17.8%。实施名牌战略，争创自治区名牌产品3个。要扶持发展中小企业。坚持抓大与促小相统一，从政策、资金、项目、技术等方面加大对中小企业的扶持力度。建立和完善政府主导、社会参与的中小企业贷款担保体系，为中小企业发展搭建信用平台，集中培育一批有发展潜力、成长能力的中小企业群体，进一步增强中小企业吸纳就业、增加税收、壮大县域经济的主力军作用。

(二)加快基础设施建设，强力推进城市化进程

着力加快市区、青铜峡、盐池、同心、红寺堡、太阳山城市建设，努力构建以大带小、整体推进、充满活力的城镇发展体系。要高起点规划城市。编制覆盖市区和青铜峡小坝的城市近期建设规划、控制性详规和修建性详规，加快修编交通、供水、消防、供热、燃气、电力、环境保护、生态建设等专项规划，完成村庄布局、特色小城镇和村庄建设规划编制。大力推行“阳光规划”，提高规划设计水平。理顺城乡规划管理体制，加强规划监管，加大规划执法力度，杜绝违法违章建设行为。要高标准建设城市。按照“拉开框架，跨过黄河，西移东扩，连为一体”的思路，推进市区高速公路以西5.2平方公里、小坝东区7.3平方公里区域内道路、供排水、供电、通讯等基础设施建设。开工建设吴忠黄河公路大桥，新修、续建金滨路向北延伸段等一批城市道路，打通市区断头路、丁字路。实施市区城市防洪排涝水利工程，推进青铜峡罗家河景观水道建设，加快水系连通步伐。实施盐池西区建设、县城出入口道路拓宽，同心县城清水河过境段及北沟综合治理、新区建设，红寺堡生态绿化等一批项目，拉开城市框架，改善城市面貌。完善提升金积、峡口、惠安堡、韦州等小城镇聚集功能。要高水平管理城市。纵深推进城乡环境综合整治暨绿化美化工程，建立长效管理机制。探索建立数字化城管模式，综合运用经济、科技等多种手段，加强市政设施、道路交通、市容市貌、社区物业等管理。不断完善社区功能，推进和谐社区建设。要高效益经营城市。培育土地、资本等要素市场，建立健全土地资源市场化配置机制，完善经营性土地招拍挂制度，搞活土地经营，盘活存量资产，提高土地资产效益。加快吴忠市国资经营集团改革步伐，拓宽城市建设融资渠道。

(三)建设十大农业产业基地，强力推进新农村建设

紧紧围绕增加农民收入，大力发展现代农业，改善农村基础设施，推进新农村建设迈出坚实步伐。要扩张优势特色产业规模。按照“一个基地一个规划、一套政策、一个班子”的要求，通过制定出台奶产业发展等政策的引导扶持作用，加快建设十大农业产业基地。全市新增优质粮食10万亩，达到70万亩；新增设施农业10万亩，达到20万亩；新增奶牛8万头，存栏达到26万头；肉牛出栏25万头；滩羊饲养量达到450万只；新增高酸苹果10万亩，达到22万亩；新增葡萄7万亩，达到18万亩；新增红枣10万亩，达到23万亩；新增人工种植甘草23万亩，达到47万亩；新增饲草30万亩，达到130万亩。要促进农产品转化增值。加大政策、项目、资金扶持力度，扶持引导各类企业和经济合作组织参与我市现代农业建设，促进产、加、销、运、储健康发展，拉长产业链。加强农村信息化建设，提高农民的网络信息应用水平。进一步创新科技特派员创业行动模式和机制，实现科技人员入户、良种良法到田。继续实施“百万农民培训”工程，培育有文化、懂技术、会经营的新型农民。积极推行农业标准化生产，加快无公害农业、绿色农业、有机农业发展。建立健全农产品质量安全监管和服务体系，逐步推行市场准入制度；加快重大动植物疫病防控体系建设和政策性农业保险扩面步伐，提高农业抗风险能力。要改善农村生产生活条件。推进村企互动共建新农村。建设农村社区。加快农村公路建设步伐，新建、改造农村公路300公里。加大农田水利建设、农业综合开发和水土治理力度，实施太阳山小河防洪、同心盐池节水补灌等项目。加快中心村与生态家园建设，加大川区沿石中高速公路两侧庄点和山区危房危窑改造力度，改造山区危房危窑3000户，新增“一池三改”农户11200户。实施农村安全饮水工程，解决20万人口安全饮水问题。要加快扶贫攻坚步伐。完成44个贫困村整村推进任务，实施生态移民工程，建设移民点5个，搬迁移民3万人，新建抗旱水窑3000眼、新型集雨场2500个，开发节水灌溉面积6万亩。大力发展劳务产业，全年输出劳务20万人以上。

(四)大力推进服务业发展升级，加快繁荣第三产业

依托自身资源特点和区位条件，按照差异化发展理念，大力发展第三产业，形成与大银川及周边城市功能互补的发展局面。要大力发展特色旅游业。加快完善旅游业发展规划，下大力气推进旅游景区基础设施配套建设，加大市场开拓和宣传促销力度，打造以青铜峡鸟岛库区为核心，辐射108塔、金沙湾、牛首山寺庙群的宁夏精品旅游区；大力发展民俗风情旅游、革命老区红色旅游、观光农业旅游、绿色生态旅游、农家乐休闲旅游等旅游项目，全年接待游客135万人次，实现旅游总收入4.6亿元。要突出发展现代商贸流通业。加速以市区国贸大厦开业为主要内容的商贸核心区建设和改造等一批市县商贸重点项目。依托市区涝河桥、同心阿伊河、盐池荣宝园等优势品牌，完善市场功能，提升牛羊肉及牛羊皮等系列附属产品集散能力。依托西市场、铁市场，全面整合废品收购市场，打造全区乃至西部可再生资源收购集散地。继续发展金融、保险、信息等现代服务业，不断拓宽服务领域。深入实施“万村千乡市场”工程，大力活跃农村消费市场。要壮大餐饮服务业。挖掘整合本地名小吃、名菜点，引导餐饮企业创新经营，提高效益，做大规模，培育知名品牌和优势企业。年内重点培育3—5家在全区叫得响的饮食名店和一批名小吃连锁店。

(五)深化改革开放和自主创新，提升经济社会发展驱动力

深入推进改革开放和自主创新，建立健全促进科学发展的体制机制，为跨越式发展提供更加强劲的动力。要继续推进改革。深化企业产权制度改革，使企业步入良性发展的轨道。推进行政管理体制改革，激活机关用人机制。加快教育教学改革，加强教师队伍建设。加快医疗卫生、科技等领域的改革。推进农村信用联社改革、农业发展银行职能转化，设立地级市邮政储蓄银行，抓好盐池农村村镇银行试点。进一步推进公共财政改革，加强财源建设，强化税收征管，规范部门预算管理，完善国库集中收付制度，扩大政府采购范围和规模，优化财政支出结构，提高财政保障能力。扎实推进农村综合改革，完成乡镇债务化解任务，启动农村义务教育债务化解工作。加快推进水权、林权改革。要提升开放水平。大力推进企业招商、园区招商、产业招商、资源招商，扩大对外经济合作，精心组织各类重大招商活动，力争在引进大型企业和重大项目上实现新突破。着力优化投资环境，加强行政、金融、商业等领域信用体系建设，严厉打击欺商、坑商等不法行为，吸引更多的企业、客商、项目、资本向吴忠聚集。年内招商引资到位资金32亿元以上。要提高创新能力。加大对科技创新的财政支持力度，探索建立科技创新基金，对技改及高新技术成果转化项目贴息。加强创新型行政管理人才和企业经营管理人才队伍建设，培养一批专业化技术人才和科技带头人。

(六)加强资源节约和环境保护，推进绿色生态建设

坚持节约优先、环保优先，强化目标管理，严格落实责任，以铁的手段推动经济发展与资源、环境相协调，增强可持续发展能力。要强化节能降耗工作。实行节能减排硬性考核。加强27家重点能耗企业的节能降耗，扩大节能减排成果。加快高耗能、高排放行业技术改造，实施昊盛、峡光等4个碱回收项目，推进电厂脱硫，新上危险废物处理设施。建设庆华循环经济示范园区，抓好金昱元、青铜峡水泥公司、金牛化肥公司等7个循环经济试点，引导企业把污染防治由末端治理转向生产全过程控制，力争培育5家市级循环经济示范企业。要加强资源节约利用。认真落实节约资源的基本国策，坚持最严格的土地管理制度，加强土地调控，坚决制止违法违纪违规用地行为，提高土地利用率。整顿规范矿产资源开发秩序，加强饮用水源保护和风能、太阳能、空中水资源利用，完善矿产资源和水资源有偿使用机制，实现有效节约利用。要加强生态环境保护。实施天然林保护、退耕还林(草)、三北防护林四期、市县环城绿化等生态绿化工程。沿S101线拓宽段等主干道路，建设绿色长廊和景观林带，打造环城宽幅林网，市区绿地面积在现有基础上翻一番。全面推进农村小康环保行动，加强农村面源污染综合整治。严格实行排放总量控制、排污许可、限期治理和环境影响评价等制度，严禁新上不符合节能环保标准的项目，坚决关闭严重浪费资源和污染环境的企业。

(七)突出改善民生，强力推进和谐社会建设

在全面提升经济发展水平的同时，更加重视改善民生。要优先发展教育事业。统筹城乡教育资源配置，促进义务教育均衡发展。巩固提高“两基”成果，确保“两基”工作顺利通过“国检”验收。全面推进素质教育，加快普及高中阶段教育；重视学前教育，关心特殊教育，发展职业教育和民族教育，引导规范民办教育，提高教育教学质量。创建7所区级示范性高中，完成市区19所城市公办学校操场标准化建设项目。要提高全民健康水平。完善新型农村合作医疗制度，健全农村三级卫生服务网络，加强城市社区卫生服务标准化、规范化建设和乡镇卫生院建设，实施吴忠市中医院迁建等一批医疗卫生基础设施项目。完善药品招标“三统一”。抓好农民健康教育与健康促进行动工程，为60周岁以上老人免费体检，儿童计划免疫实行全免费。启动创建国家卫生城市。加强人口与计划生育工作，努力实现“三无”、“一无”乡镇创建目标。要推进和谐文化建设。积极发展文化产业，健全公共文化服务体系，加快图书馆、艺术馆等文化基础设施建设。全面开展全民健身迎奥运及自治区成立50周年和地级吴忠市成立10周年系列庆祝活动。全力配合搞好50大庆献礼影片《同心》拍摄工作。要强化就业服务工作。突出抓好就业和再就业工作，全年新增城镇就业1.05万人，实现无“零就业”家庭目标。抓好职业技能培训，提高农民工劳动技能。全面推行劳动合同制度，建立工资支付保证金制度，依法维护劳动者合法权益。要完善社会保障体系。完善村干部养老保险，抓好城镇居民全民医保试点工作。积极探索失地农民、城镇失业和困难人员养老保险办法，在建筑领域和高风险行业中推行工伤保险，力争参保单位达到80%以上。加强社保基金监管，实现保值增值。稳步提高城乡居民最低生活保障标准，做好优抚安置、扶老、救孤、助残、济困等工作，规划建设流浪儿童救助保护中心。要营造稳定和谐的社会环境。全面推进依法治市进程，提高社会事务法治化管理水平。完善社会治安防控体系，加强社会治安综合治理，严厉打击违法犯罪活动，全力维护社会治安稳定。加强信访工作，畅通人民群众诉求渠道。加强法律援助工作，切实维护弱势群体合法权益。全面落实安全生产责任制，构建市县乡村四级监管网络，抓好安全领域和行业的隐患排查专项整治，坚决遏制重特大事故发生。全面规范市场经济秩序，加强价格监管，巩固产品质量和食品安全整治成果，建立长效机制，严厉打击危害人民群众健康与生命安全的非法生产经营活动和各类扰乱市场经营秩序的违法犯罪活动。认真贯彻落实党的民族宗教政策，依法妥善处理民族宗教事务，维护宗教领域和谐稳定，巩固发展平等、团结、互助、和谐的社会主义民族关系。切实做好外事侨务、防震减灾、保密等工作。加强国防教育和人防应急准备工作，深入开展双拥共建活动，加强军政军民团结。

四、加强政府自身建设，更好地服务跨越式发展

按照“为民、务实、清廉”的要求，坚持科学执政、依法执政、民主执政，以政府效能建设为突破口，全面加强政府自身建设，不断提高政府执行力和公信力，增强推进经济社会跨越式发展的能力。

努力建设法治政府。把依法行政作为政府工作的基本准则，依法决策、依法办事。重大事项没有调查研究不决策、没有专家论证不决策、没有征求群众意见不决策、没有法制部门合法性审查不决策、没有集体讨论不决策。认真贯彻《行政许可法》和《公务员法》，全面落实行政执法责任制，完善行政执法程序，细化行政裁量权，按照法定权限和程序行使权力、履行职责。探索相对集中行政处罚权，推行综合执法，切实解决多头执法和执法不公等问题。坚决防止和查处各种形式的乱检查、乱罚款、乱摊派，加强对行政执法主体、过程与结果的监督、问责，让政府部门的执法更加亲民、更加人性化。进一步完善行政复议制度，充分发挥行政复议在化解社会矛盾、解决行政争议中的作用。真正把政府管理经济社会行为纳入依法运转的轨道，做到文明执法、规范执法、公正执法，不断提高行政执法水平。

努力建设诚信政府。加强政府诚信建设，增强政府部门及公务员的诚信意识，诚信办事、诚信服务，为全社会树立诚信表率。全面落实服务承诺、首问负责、一次告知等制度，建立政府服务公开承诺、限时办结的服务方式，凡政府承诺为民办理的事，必须办结、办好；承诺支持企业、客商的优惠政策一定要全部兑现，落在实处。加强政府诚信建设的监督检查，及时纠正优惠政策兑现不到位、落实不力等现象。

努力建设高效政府。继续深化行政审批制度改革，清理、减少和规范行政审批事项，消除政策性、体制性门槛和障碍，推进行政审批向政务大厅和部门服务窗口集中，实行一门受理、优化流程、即时办理、限时办结。着力强化行政效能监督，设立行政效能投诉中心，建立政府领导督办、重大项目跟踪服务等机制，加大对重点工作、重点项目的协调和督查力度，对办事拖拉、执行不力、落实不实的责任单位和责任人，坚决问责；对失职渎职、滥用职权、吃拿卡要等行为严肃查处、决不手软。切实压缩公文，精简会议，减少政府领导事务性活动。加强公务员队伍建设，提高机关工作人员的政策水平、业务素质和办事能力。完善行政机关人才培养、使用、流动和激励机制，切实解决有事没人干、有人没事干等问题。加大机关效能与岗位责任考核力度，对不会干、不愿干、干不了事的工作人员实行责任倒查、严肃问责，在政府部门中营造更加浓厚的干事创业氛围。

努力建设服务政府。深入推进政府管理体制、服务方式的创新，强化社会管理和公共服务职能，把优化职能的着力点放到为企业、为基层、为群众搞好服务上来，完善公共服务体系，不断提高服务人民和管理社会的水平。始终带着深厚感情为民解忧、为民办事。从关系群众衣食冷暖、幸福安康的具体事抓起，从群众最期盼、最细小的事情做起，凡是群众、社会、企业需要的公共服务、公共产品都要努力去做。加强电子政务建设，坚持公开透明的办事准则，大力拓展和规范政务公开，严格公开程序，增强透明度，扩大人民群众的知情权和参与权，保障人民群众的表达权和监督权，让群众办事更方便、更快捷。

努力建设责任政府。不断强化政府部门服务经济发展、管理公共事务的责任，主动接受人大及其常委会的法律监督和工作监督，积极为政协民主监督创造条件，虚心听取对政府工作的意见和建议，不断提高办理人大代表议案、建议和政协委员提案质量。认真听取各民主党派、工商联、无党派人士和工会、共青团、妇联等人民团体的意见，强化新闻舆论和社会公众监督。建立政府信息公布制度，完善人民群众建议征集、重大事项社会公示、社会听证、专家咨询等制度，健全政府与公众沟通机制，引导和鼓励人民群众共同参与管理公共事务，切实提高行政管理透明度和公众参与度。

努力建设廉洁政府。全面落实党风廉政建设责任制，严格执行领导干部廉洁自律各项规定，加强以规范权力运行为核心的体制机制建设，着力构建教育、制度、监督并重的惩治和预防腐败体系。强化监察、审计职能，从敏感领域、敏感环节抓起，从对具体人、具体事、具体案件的调查处理入手，加大对重大项目、重点部门和土地出让、工程招投标、政府采购、产权交易等重点领域的监督力度，发现问题，坚决一追到底，依法查处。大力弘扬艰苦奋斗、勤俭节约的优良传统，节约行政资源，减少非公务性支出，努力降低行政成本。深入推进政风行风建设，坚决纠正损害群众利益的不正之风。促使每个部门、每个领导干部和公务员都正确行使权力，让人民放心，让人民满意。

各位代表，实现吴忠未来五年的发展蓝图任重道远；抓好明年的工作时不我待。让我们紧密团结在以胡锦涛同志为总书记的党中央周围，高举中国特色社会主义伟大旗帜，坚持以科学发展观统领全局，在自治区党委、政府和市委的坚强领导下，紧紧团结和依靠全市广大干部群众，继往开来，振奋精神，扎实工作，为实现吴忠跨越式发展目标而努力奋斗！
